# A Pragmatic Neuro-Rehabilitation Approach for Basilar Invagination: A Case Report

**DOI:** 10.7759/cureus.41921

**Published:** 2023-07-15

**Authors:** Anam R Sasun, Moh'd Irshad Qureshi

**Affiliations:** 1 Department of Physiotherapy, Ravi Nair Physiotherapy College, Datta Meghe Institute of Medical Sciences, Wardha, IND; 2 Neuro-Physiotherapy, Ravi Nair Physiotherapy College, Datta Meghe Institute of Medical Sciences, Wardha, IND, Wardha, IND

**Keywords:** case report, magnetic resonance imaging, tingling, pain, nprs, neurorehabilitation, basilar invagination

## Abstract

Basilar invagination is a rare pathology of the occipital bone, along with prolapsing of the vertebral column. It is a narrowing in the opening of the foramen magnum of the odontoid process. It is a well-known cause of pain and tingling in the upper limbs. However, only a few afflictions requiring physiotherapy rehabilitation in basilar invaginations have been reported. Thus, this study was carried out to investigate a case of basilar invagination. A 51-year-old female visited the neuro-outpatient department. The chief complaints of the patient were restricted overhead activities, restricted neck and shoulder movements, upper limb weakness, and tingling of bilateral upper limbs for the past two months. Clinical examination revealed pain thresholds for the neck and shoulder at nine by ten on activity and five by ten on rest. Manual muscle testing revealed a significant reduction in the strength of muscles around the neck and shoulder at three by five on bilateral upper limbs. The patient was advised to have computed tomography (CT), magnetic resonance imaging (MRI), and a bone density test to confirm the diagnosis of the condition. Investigations revealed a case of basilar invagination. But due to the financial burden, surgery couldn’t be opted for; therefore, she opted for physiotherapy rehabilitation. The patient was managed with neuro-physiotherapy rehabilitation exercises like neural tissue stretch, which included nerve gliding and nerve stretching exercises, vestibular rehabilitation exercises, and gaze stabilization exercises. The strengthening of weakened muscles was done using Delorme's technique. Cervical traction, electrotherapy, and moist heat modalities like interferential therapy and hydrocollator packs were given. It also included deep breathing exercises like diaphragmatic breathing and thoracic expansion exercises. The exercise was planned according to the frequency, intensity, time, and type (FITT) principle. Frequency: five days/week; intensity: slow to moderate pace with rest intervals; time: 60 minutes/day; type of exercise: strength training along with other exercises for a total of thirty days. The patient was able to resume her job after receiving physiotherapy rehabilitation, which played a pivotal role in decreasing her symptoms.

## Introduction

Basilar invagination is a rare pathology at the craniovertebral junction of the cervical spine. It is a condition that happens whenever the floor of the skull at the foramen magnum has grown in such a way that the superior aspect of the upper spine is more cephalad, which actually results in a constriction of the foramen magnum opening and prolapsing of the odontoid process [1]. The cause of this abnormality is either congenital or degenerative. Congenital basilar invagination may remain asymptomatic and unrecognized until adulthood. When symptoms progress and threaten disability, basilar invagination (BI) results in severe neurological, anatomical, and respiratory disturbances within the body. Clinical features of basilar invagination include headache, dizziness, loss of sensation, tingling numbness around hands and feet, weakness, nystagmus, difficulty swallowing, and restricted neck ranges [2]. Restricted neck movements (59 percent), a low hairline (48 percent), a webbed neck (57 percent), and a short neck were among the localized findings [3]. The prevalence rate of BI is less than one percent in the general population. Moreover, it is attributed to softening of the osseous structures at the base of the skull [4]. It is a rare condition that can have multiple causes. The most common causes include Chiari malformation, arthritis, syringomyelia, and Klippel-Fiel disease [5]. The diagnosis of basilar invagination is usually made through radiological investigations when the tip of the odontoid process of the cervical spine crosses the line of Chamberlain's [6]. Further diagnoses depend on the Chamberlain line, the McGregor line, and the McRae line [7]. Conservative treatments include methods like physical therapy, anti-inflammatory medication, or a neck brace. Physiotherapists cite anecdotal evidence of the clinical effectiveness of soft collars. Physiotherapy will include exercises and electrotherapy modalities.

The severity of the affliction determines how the condition can be managed. To alleviate the symptoms, treatments such as gabapentin medication and physical therapy can be used. According to our knowledge, it is one of the few case studies on the physiotherapy management of basilar invagination. The objective of this case report is to help future physiotherapists when rehabilitating patients with rare pathologies. The second objective is to provide a broader knowledge of the pathology of basilar invagination.

## Case presentation

Patient information

A 51-year-old female visited the neuro-physiotherapy outpatient department with chief complaints of restricted neck and shoulder movements, weakness, and tingling of bilateral upper limbs, along with spontaneous dizziness and headache during head movements for the past two months. Medical history revealed a history of dust allergies. Personal history revealed a history of tobacco chewing for the past seven years. Further, she revealed having fallen in her washroom three months ago. She admitted to controlling her pain using non-steroidal anti-inflammatory drugs for a few days. The physical examination was performed with the patient’s oral consent, with the back properly supported with pillows. The patient was ectomorphic. Active range of motion testing was done for the cervical and shoulder joints, which revealed decreased range of motion in all six planes of the joint. The pain, tingling sensations in the upper limbs, and dizziness got aggravated while performing neck extensions, which eventually got relieved with rest. The patient scored the pain around the neck and shoulder as nine out of ten on activity and five out of ten on rest. Grade three tenderness was present around the cervical joint. Neurological examination revealed intact pain sensations and reflexes. No facial asymmetry, or nystagmus, was seen.

Motor strength was assessed using manual muscle testing, which revealed a significant reduction in the strength of muscles around the neck and shoulder. A grade of three by five in shoulder and neck. The patient was advised to undergo radiological investigations to confirm the diagnosis of the condition. The timeline of events is illustrated in Table 1.

**Table 1:** Timeline of events

Date of events	Events
06 May 2022	An episode of a fall inside the washroom
29 July 2022	Visited Ayurveda practitioner
12 August 2022	Orthopaedic out- patient department
16 August 2022	Investigations
20 August 2022	Physiotherapy out-patient treatment day 1
18 September 2022	Last day of physiotherapy treatment

Diagnostic assessment

**Figure 1:**
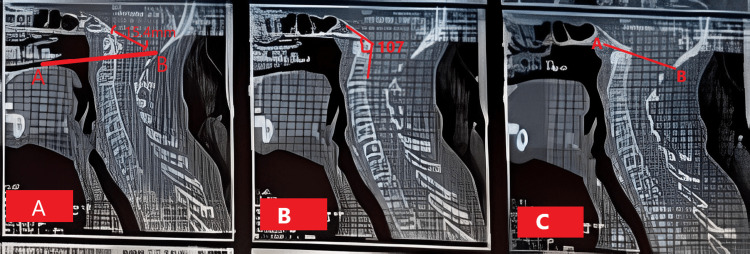
CT image of the cervical spine A: The tip of the odontoid process lies 15.4 mm above Chamberlain’s line; B: The clivus angle measures 107 degrees; C: The tip of the dens tends to transect Wackenheim’s canal and lies 2 mm below Mc Rae’s line. CT: computed tomography

These features were suggestive of basilar invagination at the tip of the odontoid process. Magnetic resonance imaging revealed disc desiccation at all cervical levels along with multilevel anterior osteophytes in the spine. Lumbar canal stenosis was noted at the lumbar 4-5 level (9.4 mm) and at the lumbar 5-sacral 1 disc level (6.2 mm). Spondylo-degenerative changes were seen in the whole spine. The patient underwent a computed tomography scan of the cervical spine, which revealed the tip of the odontoid process lies 15.4 mm above Chamberlain’s line, and the tip of the dens tends to transect Wackenheim’s canal and lies 2 mm below Mc Rae’s line. The clivus angle measures 107 degrees (Figure 1).

Therapeutic intervention

The patient wanted to resume all her overhead activities like combing, bathing, and dressing, which were hampered due to pain. The physiotherapist’s goal was to motivate the patient and make her adhere to performing all the prescribed exercises. Shoulder mobilization was given using caudal glide to improve shoulder abduction. The other goals were to improve strength and tingling sensations and make her return to her functional status. So that she can resume her job. Physiotherapy interventions are given in Table 2.

**Table 2:** Summarization of neuro-rehabilitation given to the patient Y: letter Y is used to represent the patient; reps: repetitions; L: liter; A-P: anterior-posterior; mins: minutes; IFT: interferential therapy; ADLs: activities of daily living

Sr No.	Patient goal	Physiotherapy interventions	Treatment protocol
1.	Education to the patient about her present status	Gaining consent and trust from the patient	The patient was educated about the importance of timely exercise and monitoring symptoms
2.	Mrs. Y will experience decreased pain by the end of one week	Hydrocollator and interferential therapy	Hydrocollator pack for seven minutes. IFT of frequency 100-150 Hz for five mins
3.	Mrs. Y will experience decreased tingling sensations in her upper limbs within two weeks	Nerve stretching and, nerve gliding exercises	10 reps thrice a day with a 15-second hold.
4.	Mrs. Y will be able to perform full-range shoulder and neck activities within three weeks	Shoulder Maitland mobilization, cervical traction, and active range of motion exercises	A-P, anterior glide, and caudal glides were performed to increase shoulder ranges. Two-three glides/second for 15 seconds with five reps for two weeks
4.	Mrs. Y will regain the strength of her upper limb muscles by the end of two weeks	Delorme technique	Upper limb strength training with a ½ L water bottle initially, then progressed to 1 L
5.	The patient will be able to do mild strenuous daily activities without exertion in two weeks	Diaphragmatic breathing and thoracic expansion exercises	10 reps thrice a day
6.	Mrs .Y will experience decreased dizziness by the end of three weeks	Vestibular rehabilitation exercises like gaze stabilization exercise	Mrs. Y will experience decreased dizziness by the end of three weeks. Vestibular rehabilitation exercises like gaze stabilization exercise three to times a day
7.	Decreased ADLs requiring overhead shoulder movements	Initiation and progression of overhead shoulder movements	Encourage the use of the upper limbs for overhead ADLs

Follow-up and outcome

**Figure 2:**
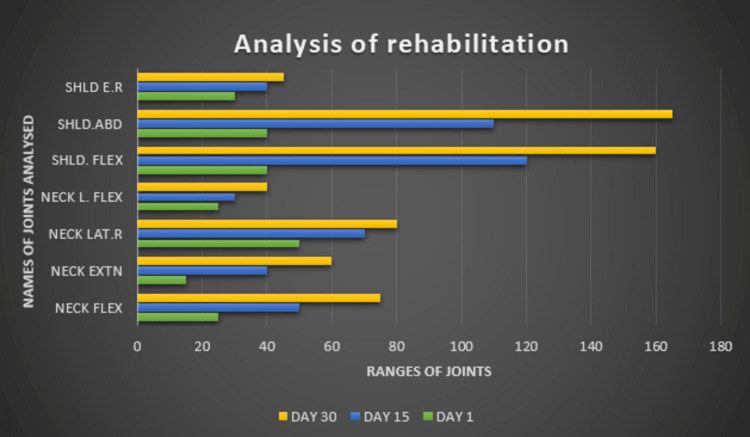
Describing the outcome analysis of rehabilitation SHLD: shoulder; FLEX: flexion; ABD: abduction; E.R: external rotation; EXTN: extension; LAT.R: lateral rotation

The numerical pain rating scale (NPRS) and joint range of motion were used as outcome measures. At the end of rehabilitation, the NPRS score was 3/10 on activity and no pain at rest. Further, there was tremendous improvement in the range of motion of joints (Figure 2). Moreover, there was a remarkable decrease in tingling sensations in the upper limbs and dizziness. There was an increase in motor strength by the end of rehabilitation; the muscle strength was 5/5 for shoulder and 4/5 for neck muscles. By the end of rehabilitation, the patient was ready to resume her work and perform all her overhead daily activities.

## Discussion

Basilar invagination is a rare neurological disorder that can occur alone and poses major treatment challenges. Basilar invagination has long been an anatomic and radiological wonderment, and it has only recently entered the realm of medicine and neurosurgery [8]. The upper part of the odontoid process and dens transfer upward towards the foramen magnum [9]. Both Chiari malformation and basal invagination were found in 6.3% and 7.6% of subjects, respectively, which is remarkably more than the general population [10]. The patient in this study complained of restricted neck and shoulder movements, along with weakness and tingling in bilateral upper limbs, for two months. After a thorough analysis of the condition, progressive week-by-week rehabilitation helped to decrease the patient's symptoms. The rehabilitation focused on increasing the range and strength of the neck and shoulder. And eliminate pain and tingling sensations in the upper limb. Muscle weakness is associated with injury as a hub for the creation of atlantoaxial instability and the subsequent development of basilar invagination. Stretching of ligaments has not been shown to play a part in the pathogenesis of basilar invagination [11]. According to Botelho et al., patients with basilar invagination are bound to have 20° greater chances of suffering from craniocervical kyphosis; it is important to look at this as a serious pathology. Moreover, head-neck flexion angulation should be investigated as a primary cause of increased basal angle [12]. According to a survey of 56 patients, the post-operative surgical results of transoral atlantoaxial reduction plate (TARP) and occipital cervical fixation have been compared, and TARP outcomes were found to be superior [13]. According to Mourad et al., It is very important for a physiotherapist to be aware of the re-growth of odontoid processes, and thus, continuous neurological examinations should be carried out periodically. According to Electricwala et al., patients with atlantooccipital assimilation do not present with signs of cervical cord compression. Therefore, they must be offered an unbiased non-operative treatment plan before considering surgical intervention.

Cervical traction, neck exercises, and posture correction can be appropriate cures for neck pain and other neck problems [14]. Other non-operative treatments like cervical interferential therapy and a rigid cervical orthosis were quite efficient in treating basilar invagination, and the patient was asymptomatic even after a long follow-up of eight months [15]. As a result, we tried to combine various approaches in order to develop and incorporate a treatment plan that will help other physiotherapists in the future to treat basilar invagination. Our patient received very good treatment-based rehabilitation, which worked to decrease the patient's pain and subsequently allowed our patient to invest more time in other associated complaints like improvement in joint movement, muscle strength, and dizziness.

There will be a few limitations to the study. First, No long-term follow-up of the study will be done. The implications of the study are that it will provide a broader context for the pathology. The future implication of the study is that this case report will provide a broader context for rare pathology.

## Conclusions

The core focus of our study was to outshine the relevant concepts about a rare pathology of basilar invagination. The study highlights the importance of conservative physiotherapy rehabilitation, which works to treat the symptoms. Physiotherapy rehabilitation proved effective in treating symptoms of basilar invagination. The case report highlights a comprehensive, week-by-week integrative rehabilitation protocol to treat basilar invagination. By the end of rehabilitation, the majority of the goals had been achieved, including improved cervical and shoulder ranges, decreased pain, reduced tingling sensations in the upper limbs, and decreased dizziness during neck extension activities. Our patient was able to resume her job after receiving physiotherapy rehabilitation.
